# Accuracy of planar anteversion measurements using anteroposterior radiographs

**DOI:** 10.1186/s12891-019-2979-0

**Published:** 2019-12-05

**Authors:** Gwang Chul Lee, Sang Hong Lee, Sin Wook Kang, Hyung Seok Park, Suenghwan Jo

**Affiliations:** Department of Orthopaedic Surgery, Chosun University Hospital, 365 Pilmundae-ro, Dong-gu, Gwangju, 61453 Republic of Korea

**Keywords:** Anteversion, Inclination, Hip-centered anteroposterior, Pelvis anteroposterior, Total hip replacement

## Abstract

**Background:**

Several methods using simple anteroposterior (AP) radiographs have been suggested for the measurement of anteversion of the cup component after total hip arthroplasty. Herein, we compared six widely used anteversion measurement methods using two different types of AP radiograph, the conventional pelvis AP and hip-centered AP radiographs, to identify the measurement method and the type of radiograph that would provide the highest accuracy and reliability.

**Methods:**

We developed two custom-made bi-planar anteversion measurement models for the validation test. The models were designed for pelvis AP and hip-centered AP radiographs, respectively. The radiographs were acquired using the inclination angles of both models, changing from 10° to 70° at 10° increments. For each inclination angle, anteversion was changed from 0° to 30° at 5° increments. The measurements were obtained independently by two orthopedic surgeons blinded from each other’s measurements, using the methods of 1) Pradhan et al., 2) Lewinnek et al., 3) Widmer et al., 4) Liaw et al., 5) Hassan et al., and 6) Ackland et al. The measurements were repeated after 2 months. The accuracy, compared with that of the reference angle, and intra-observer and inter-observer reliabilities of each method were calculated.

**Results:**

The highest accuracy was found when the method of Liaw et al. was used with hip-centered AP radiographs, which showed a difference of 1.37° ± 1.73 from the reference angle. Moreover, regardless of the type of radiograph, the methods by Pradhan et al., Lewinnek et al., and Liaw et al. showed excellent correlations with the reference anteversion. However, substantial differences were found when the methods by Widmer et al., Hassan et al., and Ackland et al. were used, regardless of the type of radiograph used. When anteversion was measured in an inclination between 30° and 50°, the method of Pradhan et al., when used with pelvis AP radiographs, showed the highest accuracy (1.23° ± 0.92°). We also found no significant difference in anteversions between the measurements made on pelvic and hip-centered AP radiographs. Both interobserver and intraobserver reliabilities were high for all the measurements tested.

**Conclusions:**

The methods by Pradhan et al., Liaw et al., and Lewinnek et al. may provide relatively accurate anteversion measurements with high reliability, regardless of the type of radiograph.

## Background

Accurate positioning of the implanted prosthesis after total hip replacement is critical to achieve optimal outcomes, as postoperative complications including polyethylene liner wear, impingement, and instability are largely attributed to malposition of the acetabular cup [1–5]. The appropriateness of the acetabular cup position is determined by measuring inclination and anteversion. While measurement of inclination is relatively straightforward and can be conducted using simple pelvis anteroposterior (AP) radiographs, controversies remain regarding the measurement of anteversion of the acetabular component.

Measurement of anteversion using a cross-table hip lateral view is one of the most commonly used methods [2, 6, 7]. However, this method may provide inaccurate measurements in patients with joint contracture or lumbar stiffness or if the hip lateral radiograph has been inadequately acquired [8–10]. As such, a number of methods have been suggested to measure anteversion using simple AP radiographs, which includes pelvic AP and hip centered AP, and several studies have validated their accuracy and reliability [10–12]. However, the anteversion measurement formula that provides the most accurate anteversion measurement remains controversial.

The current study compared six widely used anteversion measurement methods using two different radiographs (conventional pelvis AP vs. hip-centered AP) to determine the measurement method and the type of radiograph that provides the highest accuracy and reliability. More specifically, we aimed to determine 1) the measurement method and the type of radiograph that reveals anteversion that is closest to the actual cup anteversion and 2) whether anteversion measurements using the suggested methods are reliable. We developed a custom-made bi-planar anteversion measurement model for validation.

## Methods

**Fig. 1:**
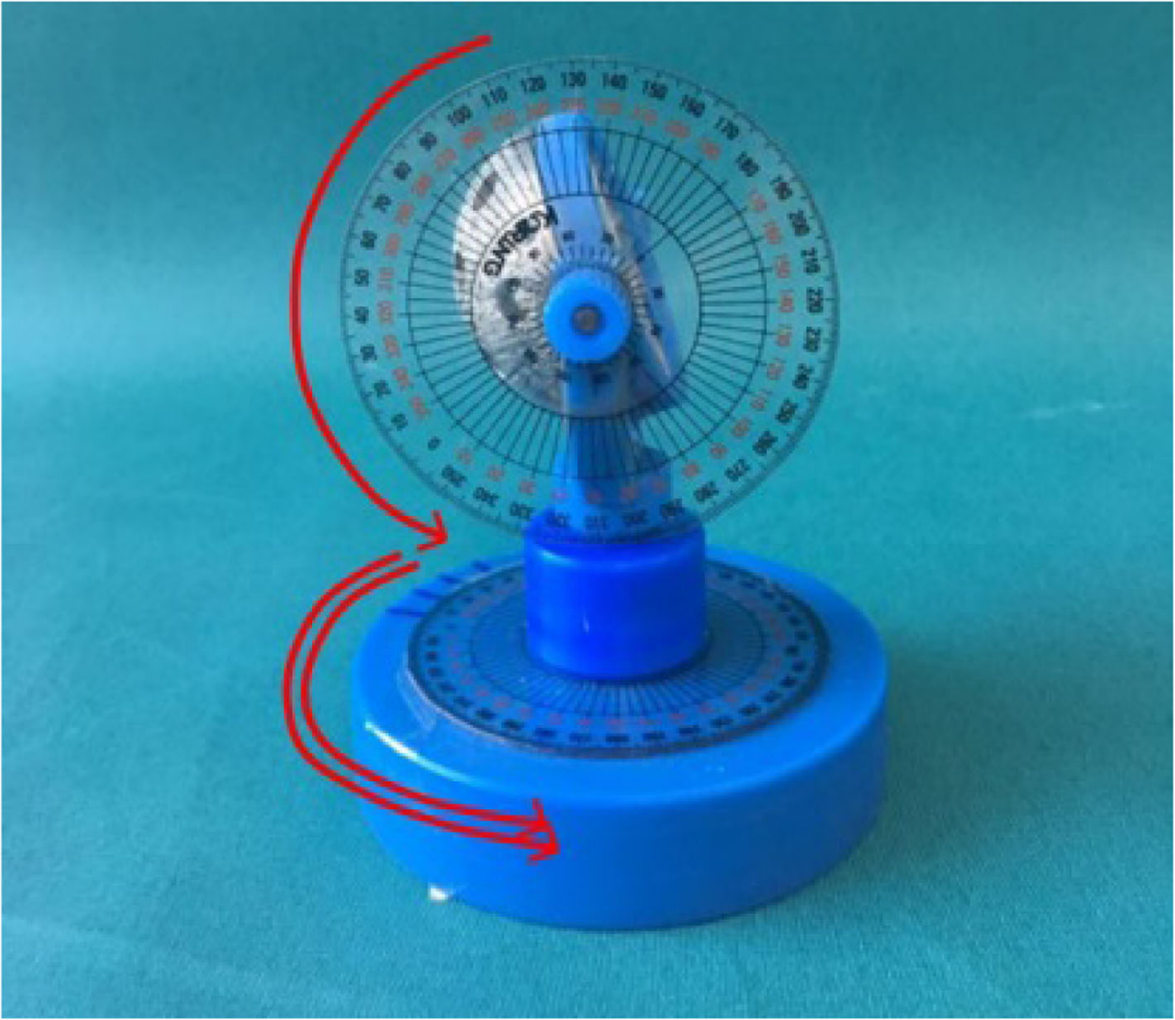
Custom-made bi-planar anteversion measurement device allowing inclination and anteversion of the mounted cup with protractors in two axes. The solid arrow indicates the change in anteversion, while the hollow arrow indicates the change in inclination

**Fig. 2:**
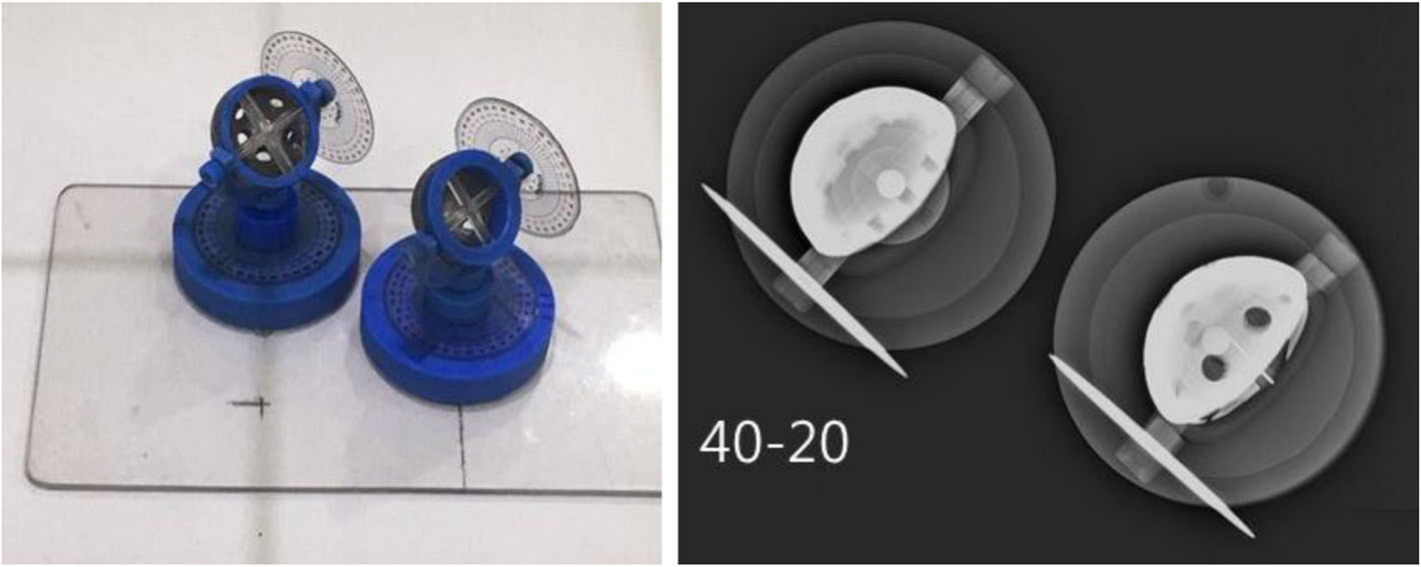
Two custom-made bi-planar anteversion measurement models fixed to plexiglass. The distance between two models represents the distance from the center of the triangle formed by both anterior superior iliac spine (ASIS) and symphysis pubis and the hip joint

A 54-mm acetabular cup (Trilogy, Zimmer, Indiana, USA) was attached to a custom-made bi-planar anteversion measurement model that enabled control of inclination and anteversion (Fig. 1). The model had two axes that represented inclination and anteversion, respectively, and a goniometer was attached to each axis for precise control of changes in both inclination and anteversion. The cup was fixed to the plexiglass plate at a 10-cm height to represent the normal height of the hip joint in the supine position (Model A). Another model with the same cup and design was manufactured (Model B) and was fixed to the plexiglass plate 9.9 cm lateral and 4.9 cm distal to the Model A (Fig. 2). The distance between the two models represented the distance from the center of the triangle formed by the anterior superior iliac spine (ASIS) and symphysis pubis, which is typically used for conventional pelvis AP radiographs, and the hip joint. Thus, an X-ray beam directed toward Model A represented the simple X-ray in hip-centered AP radiographs, while the image in Model B represented the acetabular cup in conventional pelvis AP radiographs. The radiographs were acquired with both models’ inclinations changing from 10° to 70° at 10° increments; for each inclination angle, the anteversion was changed from 0° to 30° at 5° increments. Therefore, X-rays of the two models were acquired in 49 scenarios.

All images were digitally acquired using a Picture Archiving and Communication System (INFINITT PACS system, Seoul, South Korea), and all measurements on radiographs were subsequently conducted using PACS software. The measurements were conducted independently by two orthopedic surgeons blinded from each other using six methods described by Pradhan et al. [13], Lewinnek et al. [1], Widmer et al. [14], Liaw et al. [15]. Hassan et al. [16], and Ackland et al. [17], respectively. Before measuring anteversion, the two evaluators held a consensus-building session and clarified the definitions for each measurement method. All measurements were blinded from each other, and the measurements were repeated after 2 months to calculate intra-observer correlations.

### Anteversion measurement methods (Fig. 3)

**Fig. 3:**
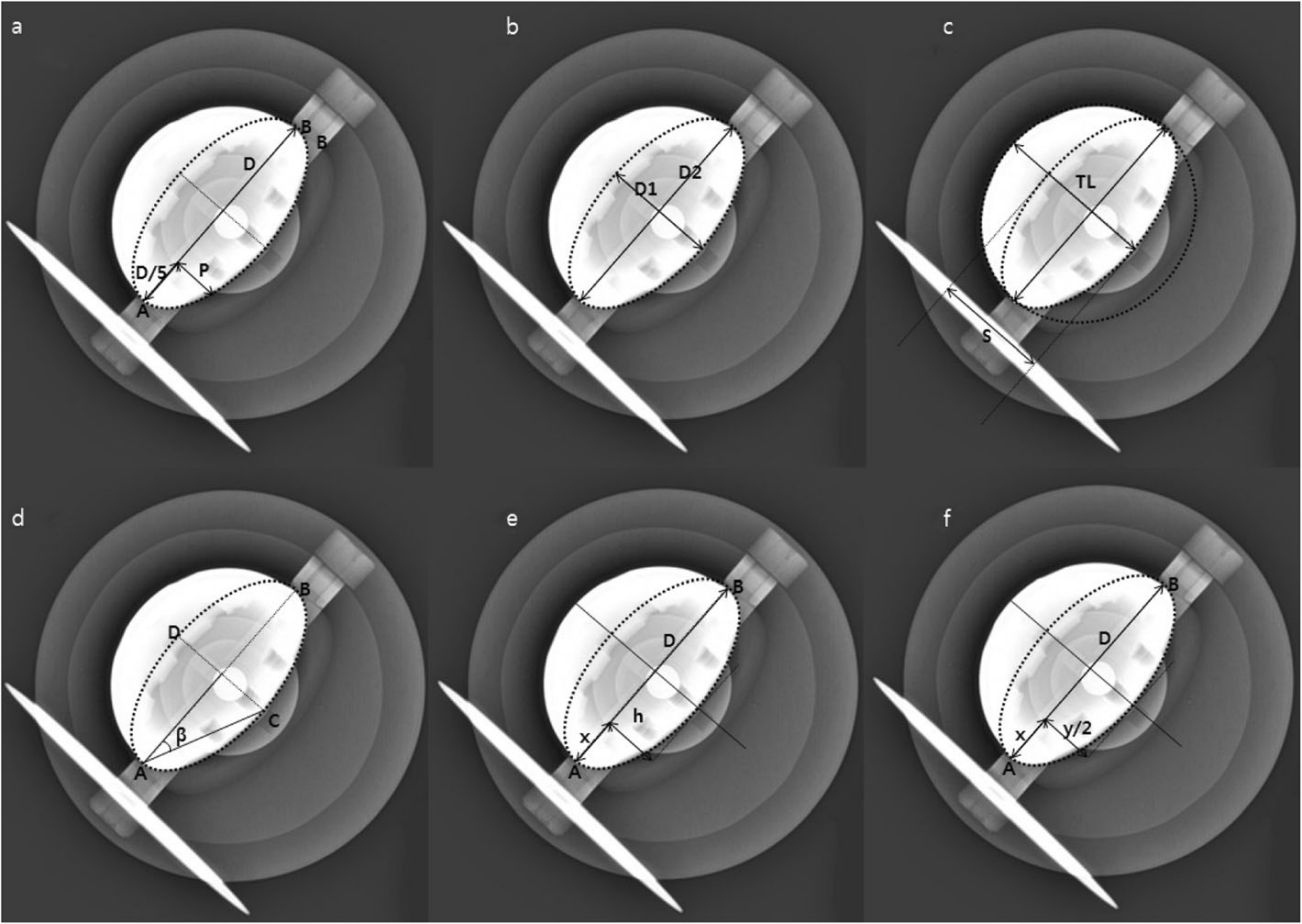
Methods for measuring anteversion on plane anteroposterior radiographs. a Pradhan et al. (arcsin (p/0.4D)), b Lewinnek et al. (arcsin (D1/D2)), c Widmer et al. (arcsin([S]/[TL]), d Liaw et al. (sin-1 tan β), e Hassan et al. ([arcsin [(h/D) / √([m/D] – [m2/D2])]], f Ackland et al. (arcsin [2y / 2√(2Dx – x2)])

1. Pradhan et al.’s method [13] = arcsin (p/0.4D) (Fig. 3a)

In which *D* is the maximum distance across the long axis of the ellipse of the component. A line is drawn perpendicular to the long axis and intersecting the rim of the component beginning at a point one-fifth of the total distance of the longitudinal line, and *p* is the distance along this perpendicular line from the longitudinal line to the rim.

2) Lewinnek et al.’s method [1] = arcsin (D1/D2) (Fig. 3b)

In which *D1* is the distance across the short axis of an ellipse drawn perpendicular to the long axis of the acetabular component and *D2* is the distance of the long axis, which is considered the maximal diameter of the implant.

3) Widmer et al.’s method [14] = arcsin ([S]/[TL]) (Fig. 3c)

In which *S* is the short axis of the ellipse and *TL* is the total length of the projected cross-section of the component along the short axis. This method shows a linear correlation for values of *S/TL* between 0.2 and 0.6.

4) Liaw et al.’s method [15] = sin-1 tan β (Fig. 3d)

In which β is the angle formed by the long axis of the component (the line from point A to B), line connecting the top point of the ellipse, and endpoint of the long axis (the line from point A to C).

5) Hassan et al.’s method [16] = [arcsin [(h/D) / √([m/D] – [m^2^/D^2^])] (Fig. 3e)

In which *D* represents the maximum diameter of the acetabular component, *m* is the distance along *D* that is not obscured by the femoral head, and *h* is the length of the perpendicular line dropped from the endpoint of the distance *m* to the acetabular rim.

6) Ackland et al.’s method [17] = arcsin [2y / 2√(2Dx – x^2^)] (Fig. 3f)

In which *D* is the distance of the long axis of the acetabular component and *x* is the distance along the line *AB*. An arbitrary tangent is drawn at a right angle to the diameter, and *y* is the distance from the two-cup rims along this tangent.

### Statistics

Reliability was defined as the consistency in the measurements, while accuracy was defined as the proximity to the reference anteversion angle. The reference anteversion was defined as the anteversion measured by the protractor of the custom-made bi-plane anteversion measurement model. The statistical analysis was performed using SPSS for Windows version 22.0 software (SPSS Inc., Chicago, Illinois). For the assessment of reliability based on inter- and intra-observer measurements, the intraclass correlation coefficient (ICC) and 95% confidence interval were calculated using the two-way random effects model assuming a single measurement and absolute agreement. ICC values were characterized as slight (0.00 to 0.20), fair (0.21 to 0.40), moderate (0.41 to 0.60), substantial (0.61 to 0.80), and almost perfect (> 0.80) [18]. For the assessment of accuracy, mean differences from the anteversion measurements of each method and reference anteversion were calculated and presented as mean ± standard deviation. To measure accuracy of each methods as compare to the reference anteversion, the paired T-test and Pearson’s correlation coefficients were used with 0.00 to 0.20 representing poor, 0.21 to 0.40 representing fair, 0.41 to 0.60 representing moderate, 0.61 to 0.80 representing good, and 0.81 to 1.00 representing excellent [18]. Student’s T-test and Bland-Altman plots were utilized to find discrepancies between the measurements obtained using conventional pelvis AP and that using hip-centered AP radiographs. Statistical significance was set at *p* < 0.05.

To assess the bias resulting from including outliers in the data, subset analysis for the accuracy of anteversion was performed for anteversion within a safe inclination zone (30° to 50°).

## Results

Inter- and intra-observer correlations (based on the ICC) were almost perfect in all measurements (Table 1). The smallest difference from the reference anteversion was observed using Liaw et al.’s method in hip-centered AP radiographs, which showed a difference of 1.37° ± 1.73 from the reference. Moreover, regardless of the radiograph type, the methods by Pradhan et al., Lewinnek et al., and Liaw et al. showed relatively small, non-significant differences from the reference anteversion (*p* > 0.05). However, significant differences were observed using the methods by Widmer et al., Hassan et al., and Ackland et al. regardless of the radiograph used (Table 2). When the anteversions were measured in the inclination between 30° and 50°, Pradhan et al.’s method in pelvis AP radiographs showed the smallest difference (1.23° ± 0.92°) (Table 3). Regardless of the radiographic method, anteversion measured using Pradhan et al.’s, Lewinnek et al.’s, and Liaw et al’s methods had excellent correlations compared with the reference anteversion, while those of Widmer et al., Hassan et al., and Ackland et al. had good to moderate correlations (Table 4).

**Table 1:** Inter-observer and intra-observer reliabilities of measurement

Measurement method	Inter-observer reliability		Intra-observer reliability	
	ICC	95% CI	ICC	95% CI
Pradhan et al. [13]	0.938	0.901–0.958	0.934	0.897–0.952
Lewinnek et al. [1]	0.937	0.897–0.967	0.916	0.845–0.974
Widmer et al. [14]	0.928	0.884–0.956	0.933	0.866–0.966
Liaw et al. [15]	0.887	0.835–0.906	0.908	0.839–0.929
Hassan et al. [16]	0.902	0.873–0.937	0.899	0.879–0.941
Ackland et al. [17]	0.886	0.847–0.923	0.913	0.868–0.933

*ICC* intraclass correlation coefficient, *CI* confidence interval

**Table 2:** Differences between measured and reference anteversions in pelvis and hip-centered AP radiographs

Measurement method		Mean ± SD difference from reference anteversion	Maximum difference from reference anteversion	*p* value
Pradhan et al. [13]	Pelvis AP	1.62 ± 1.54	5.43	0.845
	Hip-centered AP	−3.11 ± 1.14	−4.83	0.629
Lewinnek et al. [1]	Pelvis AP	2.84 ± 1.59	6.61	0.358
	Hip-centered AP	−2.91 ± 1.56	−5.34	0.521
Widmer et al. [14]	Pelvis AP	−7.10 ± 3.96	−14.46	0.061
	Hip-centered AP	−15.66 ± 3.80	−21.13	< 0.001
Liaw et al. [15]	Pelvis AP	2.52 ± 1.59	6.42	0.763
	Hip-centered AP	1.37 ± 1.73	4.31	0.692
Hassan et al. [16]	Pelvis AP	− 12.95 ± 9.71	−33.97	< 0.001
	Hip-centered AP	−18.11 ± 7.59	−30.9	< 0.001
Ackland et al. [17]	Pelvis AP	−10.15 ± 6.73	− 24.24	< 0.001
	Hip-centered AP	−13.87 ± 5.48	−28.89	< 0.001

*AP* anteroposterior, *SD* standard deviation

**Table 3:** Differences between measured and reference anteversions in pelvis and hip-centered AP radiographs within a safe inclination zone (30 °to 50 ° )

Measurement method		Mean ± SD difference from reference anteversion	Maximum difference from reference anteversion	*p*-value
Pradhan et al. [13]	Pelvis AP	1.23 ± 0.92	2.90	0.429
	Hip-centered AP	−2.66 ± 1.14	−4.83	0.752
Lewinnek et al. [1]	Pelvis AP	2.46 ± 0.98	3.92	0.235
	Hip-centered AP	−2.65 ± 1.12	−4.69	0.367
Widmer et al. [14]	Pelvis AP	−7.61 ± 3.74	−14.46	< 0.001
	Hip-centered AP	−14.99 ± 4.12	−20.64	< 0.001
Liaw et al. [15]	Pelvis AP	2.13 ± 0.96	3.35	0.523
	Hip-centered AP	1.76 ± 1.55	4.31	0.847
Hassan et al. [16]	Pelvis AP	−11.65 ± 8.92	−29.35	< 0.001
	Hip-centered AP	−17.61 ± 8.04	−29.42	< 0.001
Ackland et al. [17]	Pelvis AP	−10.84 ± 6.98	−24.22	< 0.001
	Hip-centered AP	−13.56 ± 5.30	−28.89	< 0.001

*AP* anteroposterior, *SD* standard deviation

**Table 4:** Accuracy of each anteversion measurement method compared to reference anteversion using Pearson’s correlation

		Correlation coefficient	*p* value
Reference			
Pradhan et al. [13]	Pelvis AP	0.958	< 0.001
	Hip-centered AP	0.973	< 0.001
Lewinnek et al. [1]	Pelvis AP	0.962	< 0.001
	Hip-centered AP	0.968	< 0.001
Widmer et al. [14]	Pelvis AP	0.851	< 0.001
	Hip-centered AP	0.791	< 0.001
Liaw et al. [15]	Pelvis AP	0.964	< 0.001
	Hip-centered AP	0.969	< 0.001
Hassan et al. [16]	Pelvis AP	0.575	< 0.001
	Hip-centered AP	0.610	< 0.001
Ackland et al. [17]	Pelvis AP	0.774	< 0.001
	Hip-centered AP	0.779	< 0.001

*AP* anteroposterior

**Fig. 4:**
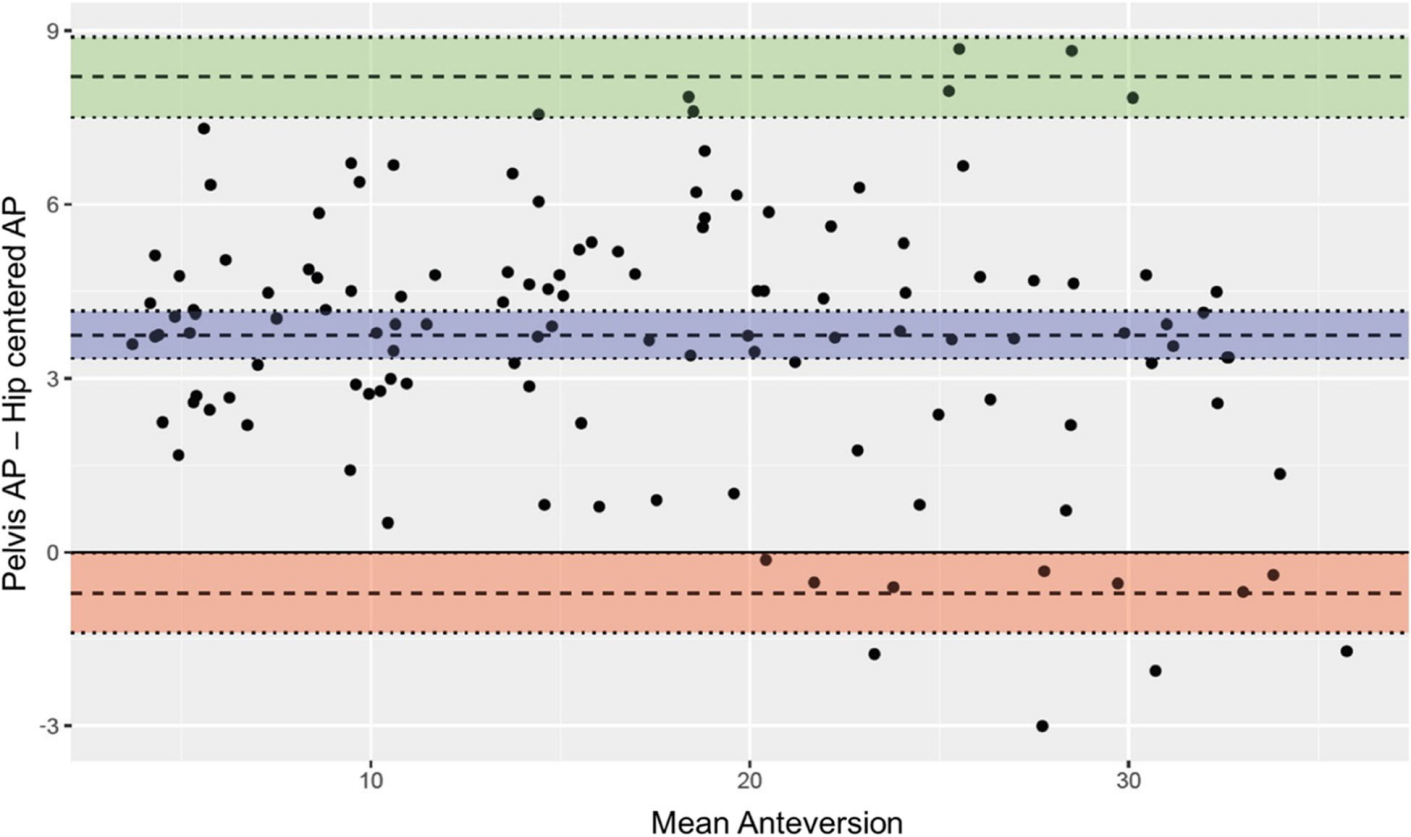
Bland–Altman plots showing differences between anteversion measurements using pelvis AP and hip-centered AP radiographs

When comparing anteversion measured according to the type of the radiographs, the Bland-Altman plots show that there is approximately 3 degree differences between the measurements made in pelvis AP as compare to that made in hip centered AP (Fig. 4). Also, Table 2 shows that there was a tendency toward smaller differences to reference anteversion when the measurement was performed using pelvis AP radiographs. However, no significant difference was found between the two radiographs when measurements were compared in all methods (*p* > 0.05 in all methods).

## Discussion

An important finding of our study is that the anteversion measurement methods described by Pradhan et al., Lewinnek et al., and Liaw et al. were able to provide anteversions with mean differences from the reference angle of less than 4° from the actual anteversion and were able to show high reliability. Moreover, measurements using hip-centered AP radiographs did not show superior accuracy compared to those using conventional AP radiographs; therefore, additional hip-centered AP radiographs are not recommended to improve the accuracy of anteversion measurements.

Correct positioning of the acetabular cup plays a significant role in minimizing wear and maintaining stability. The recommended inclination and version of the acetabular cup are controversial, but the literature often refers to inclinations between 30° and 50° and anteversion between 5° and 30° as “safe zones” [1, 19–21]. Therefore, measuring inclination and anteversion following total hip arthroplasty is essential in order to predict outcomes following the surgery. The inclination angle of the acetabular cup is defined as the angle formed by the tear drop line and elliptical long axis of the entrance of the acetabular cup; thus, the inclination angle can be directly measured using simple AP radiography [14]. However, anteversion of the acetabular component may be less accurate and difficult to obtain using two-dimensional simple radiographs [10, 11].

Due to the potential limitation of measuring anteversion in the cross-table hip lateral view, a number of studies have attempted to accurately measure the positions of the acetabular components in plain AP radiographs using complex mathematics and trigonometric functions to describe the ellipses of the acetabular cup boundaries [1, 13–17]. These methods have been validated in many studies; however, we believe that there are several limitations in how previous studies have validated anteversion measurement methods. First, many previous studies used computed tomography (CT) axial scans to validate the anteversion measured using plain AP radiographs [22–26]. However, this may cause potential bias as the anteversion measured on CT scans reflects anatomical anteversion, while the reference anteversion utilized in previous anteversion measurement methods using simple radiographs varied. Second, anteversion measurements may show considerable differences depending on how the simple AP radiograph is acquired. In simple pelvis AP radiographs, the radiation beam is projected toward the center of the triangle formed by the ASIS and symphysis pubis. Thus, the radiation beam received by the hip joint in simple pelvis AP radiographs is deviated by about 6°. In contrast, the hip joint receives a perpendicular radiation beam in hip-centered AP radiographs, and therefore, it can be hypothesized that there would be up to 6 degrees of difference depending on how the X-ray is acquired. Most previous reports did not specify where the center of the beam was directed or the reference plane that was utilized. The type of radiograph and reference plane for how anteversion was defined in the original articles are described in Table 5.

**Table 5:** Reference radiographs and planes in previous literature measuring anteversion in plain AP radiographs

Measurement method	Type of radiograph	Reference plane
Pradhan et al. [13]	Hip-centered AP	Planar
Lewinnek et al. [1]	Not specified	Not specified
Widmer et al. [14]	Pelvis AP	Planar
Liaw et al. [15]	Hip-centered AP	Planar, True
Hassan et al. [16]	Hip-centered AP	Planar
Ackland et al. [17]	Hip-centered AP	Planar, True

*AP* anteroposterior

Many studies have attempted to validate the accuracy and reliability of proposed methods of measuring anteversion using plain AP radiographs but have revealed inconsistent results [10–12]. Marx et al. compared five proposed formulas (Pradhan et al., McLaren et al., Hassan et al., Ackland et al., and Widmer et al.) to measure anteversion using AP radiographs utilizing a CT-based navigation system as a reference [11]. They concluded that all five formulas yield substantial differences in anteversion angles. Nho et al. compared six formulas (Lewinnek et al., Widmer et al., Hassan et al., Ackland et al., Liaw et al., Woo and Morrey et al.) on CT axial scans, reporting that the methods by Lewinnek et al., Hassan et al., Liaw et al., Woo et al., and Morrey et al. provided satisfactory results [11]. Nomura et al. compared five formulas (Lewinnek et al., Widmer et al., Liaw et al., Pradhan et al., Woo and Morrey et al.), concluding that the values from Widmer et al.’s method were most similar to those measured using CT [12]. It should be noted that the study by Nomura et al. is the only study to have utilized the functional coronal plane as a reference, while the other two studies used CT axial scans to measure anteversion [11]. The study by Lu et al. compared anteversion as measured by Lewinnek et al. with three-dimensional CT scans and concluded that Lewinnek et al.’s method is reliable and accurate [27]. The result of the current study shows that, while all measurements had high reliability, the accuracy was high only in the methods by Pradhan et al., Lewinnek et al., and Liaw et al. We observed significant differences from the reference anteversion values for the methods by Widmer et al., Hassan et al., and Ackland et al. We were unable to identify the reasons why the results of our study differed from those of previous studies; however, we believe that we added precision to the reference anteversion values by adding a goniometer to the bi-planar model.

We also found no significant difference in comparisons of anteversion measurements between conventional pelvis and hip-centered AP views. In general, measurements obtained using pelvis AP radiographs tended to show values closer to the reference. However, the difference was minimal and insignificant. Therefore, we suggest that additional hip-centered AP radiographs are not recommended to improve the accuracy of anteversion measurements.

Another finding of our study was that the anteversion measurements tended to be closer in value to the reference anteversion in the inclination between 30° to 50°. As most of the cup during total hip arthroplasty is targeted in this range, the methods by Liaw et al., Pradhan et al., and Lewinnek et al. can be used with relatively high accuracy if the inclination is not excessively malpositioned.

Compared to previous studies, our study showed excellent reliability in all methods. We believe this was demonstrated because we did not utilize X-rays acquired from a human pelvis. As our model did not include soft tissue or a metal femoral head, we were able to identify the boundaries of the reference variables precisely. Nevertheless, we acknowledge that this may also be a potential limitation of the current study since the accuracy may be lower when measured in X-rays of actual patients who have undergone total hip arthroplasty due to the interference of the soft tissue and metallic head, which may result in image artifacts in the radiograph. Another limitation of the current study is that we used only one type of cup. Thus, our results may only be applied to a cup that is perfectly hemispherical.

Nevertheless, we were able to validate the accuracy of the cup component anteversion measurement method precisely by applying custom-made bi-planar anteversion measurement models. We also believe that this is the only study that has explored whether hip-centered AP radiographs for the purpose of measuring anteversion are necessary.

## Conclusions

The results of the present study indicate that the methods by Pradhan et al., Liaw et al., and Lewinnek et al. may provide high accuracy in measuring cup anteversion regardless of the radiograph used. We recommend using these three methods for measuring anteversion of the cup in conventional pelvis AP radiographs.

## Data Availability

The datasets used and/or analyzed during the current study are available from the corresponding author on reasonable request.

## Abbreviations

AP: Anteroposterior
ASIS: Anterior superior iliac spine
CT: Computed tomography
ICC: Intraclass correlations
